# Rediscovery and identity of *Pumilomyia protrahenda* De Stefani (Diptera, Cecidomyiidae) in Sicily with redescription and reassessment of its taxonomic position

**DOI:** 10.3897/zookeys.617.9850

**Published:** 2016-09-15

**Authors:** Marcela Skuhravá, Bruno Massa, Giuliano Cerasa

**Affiliations:** 1 Bítovská 1227/9, 140 00 Praha 4, Czech Republic; 2 Department of Agriculture and Forest Sciences, University of Palermo, Viale Scienze bd 5A, 90128 Palermo, Italy

**Keywords:** Rhopalomyia protrahenda, gall midge, Artemisia arborescens, Italy

## Abstract

A population of the gall midge *Pumilomyia protrahenda* De Stefani, 1919 causing galls on *Artemisia arborescens* (Asteraceae) was discovered near Palermo (Sicily) in 2008. This species had not been found since 1918. Detailed study of morphological characters of adults, larvae and pupae revealed that *Pumilomyia protrahenda* belongs to the genus *Rhopalomyia* Rübsaamen, 1892, tribe Rhopalomyiini. The monotypic genus *Pumilomyia* De Stefani, 1919 is therefore synonymized under *Rhopalomyia* Rübsaamen, 1892. *Rhopalomyia protrahenda* **comb. n.** is redescribed, with important morphological characters illustrated. Adults have one-segmented palpi, antennae with 12–13 short flagellomeres and long legs with simple tarsal claws. A neotype is designated in the present paper because the type of this species is lost. The host plant has a circum-Mediterranean distribution but the gall midge is currently known only from Sicily, where it completes several generations between January and May.

## Introduction

De Stefani (1919) found galls on *Artemisia arborescens* L., reared adults, established a new genus *Pumilomyia*, and described male, female, pupa, larva and egg as a new gall midge species, *Pumilomyia protrahenda*, together with a photograph of its gall. He included this new genus in the group Asphondylariae (now: Asphondyliini). In his publication he expressed his doubt that this genus really belonged to this group because it differed from other representatives in some morphological characters. The genus *Pumilomyia* (Cecidomyiidae) with its type species *Pumilomyia protrahenda* is included in the family Cecidomyiidae (Skuhravá 1986, 2006) and in the list of gall midges of Italy (Skuhravá and Skuhravý 1994) and Sicily (Skuhravá et al. 2007).

Remarkably, this species was known only from De Stefani’s original description in 1919 more than 90 years ago. In recent years we have searched for the galls of this species at its type locality in the Botanical Garden in Palermo, where De Stefani found galls and also in its surroundings but without success. In March 2008 we discovered galls of *Pumilomyia protrahenda* on leaves of *Artemisia arborescens* at Mt. Raffo Rosso (Sicily), west of Palermo.

This recent find of larvae, pupae and adults is important because the De Stefani’s collection, including many type specimens of species described by him, is considered to be lost (V. Caleca, pers. comm.). We take this opportunity to redescribe the species and designate a neotype.

## Materials and methods

Material for this study was collected at three localities in the area of Palermo (Sicily) in 2008, 2012, 2015 and 2016. Branches of *Artemisia arborescens* with galls attributed to *Pumilomyia protrahenda* were transferred to the laboratory at the Palermo University and placed in rearing cages where they were maintained at ambient temperature to obtain adults. In the field we bagged branches of *Artemisia arborescens* including galls with fine mesh tulle. Adults which emerged from galls and immature stages that were dissected from galls were preserved in 70% ethanol for subsequent morphological studies. Specimens were examined through a stereomicroscope Wild-Heerbrugg M8. A series of images was taken using a Leica DM2500 compound microscope and a Leica DFC450C mounted camera with Leica Application Suite software. Some galls were photographed with a Canon 7D digital camera provided with a Canon MP-E 65 mm macrolens and photos were integrated using the freeware CombineZP (Hadley 2011). Some adults, larvae and pupae were mounted on microscope slides in Canada balsam and some in Hoyer´s medium. Part of the material is preserved in the collections of B. Massa and G. Cerasa, University of Palermo, Department of Agriculture and Forest Sciences, Palermo, Sicily (BMUP), and microscope slides of gall midges as well as several adults preserved in 70% ethanol are in the collection of M. Skuhravá, National Museum, Praha, Czech Republic (NMPC).

## Taxonomy

### Rhopalomyia protrahenda

Taxon classificationAnimaliaDipteraCecidomyiidae

(De Stefani, 1919) comb. n.

Pumilomyia protrahenda De Stefani 1919: 72.
Pulmilomyia artemisiae De Stefani 1929: 262.
Pumilomyia artemisiae De Stefani 1929: 263, 264.

#### Redescription.

**Figures 1–11.**
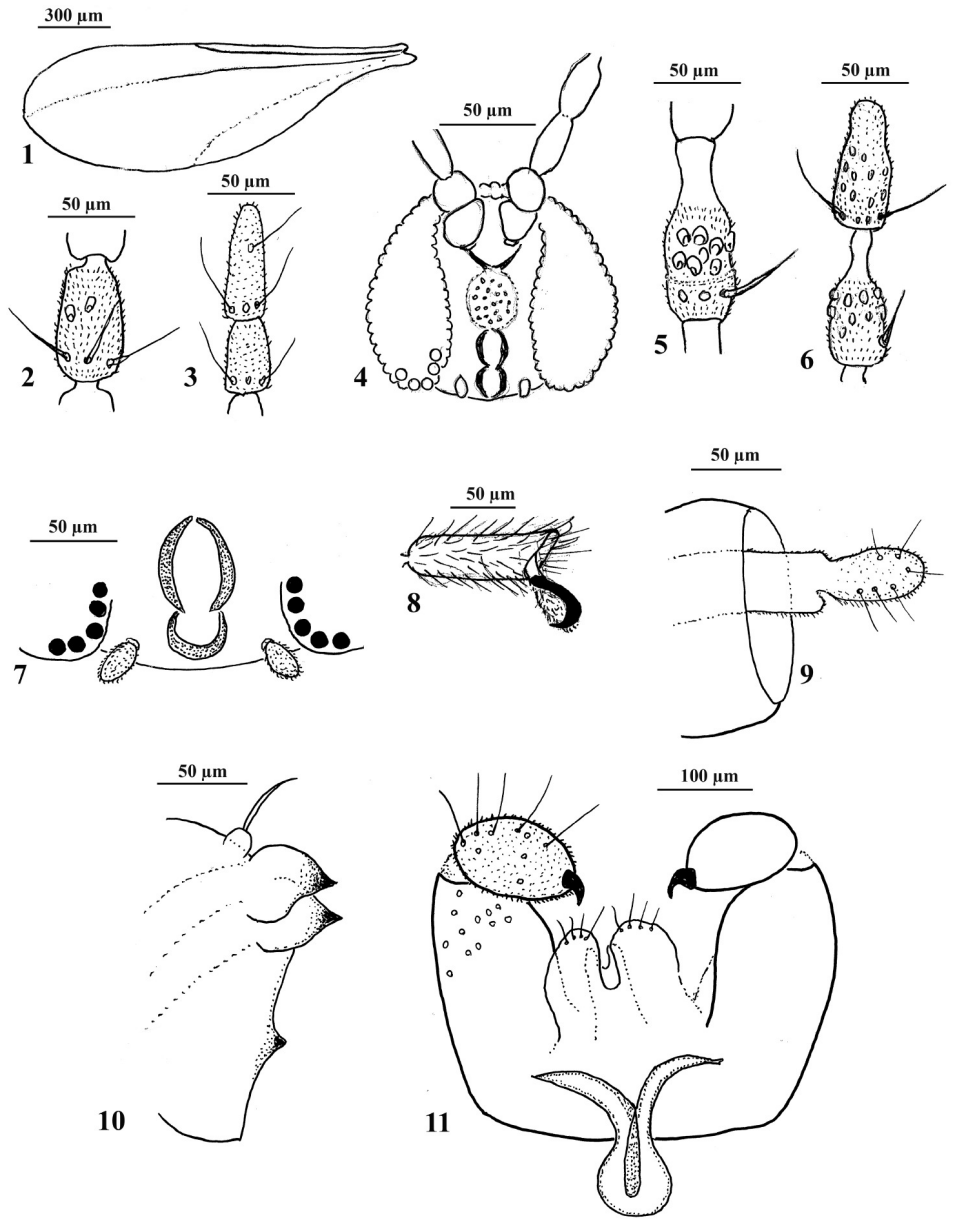
*Rhopalomyia protrahenda* comb. n. **1** Females wing **2** female, fifth flagellomere **3** female, two apical flagellomeres **4** female, head **5** male, fifth flagellomere **6** male, two apical flagellomeres **7** female, mouthparts **8** fifth tarsomere with simple claw and empodium **9** terminal part of ovipositor **10** pupal head, lateral view **11** male terminalia.

**Figures 12–19.**
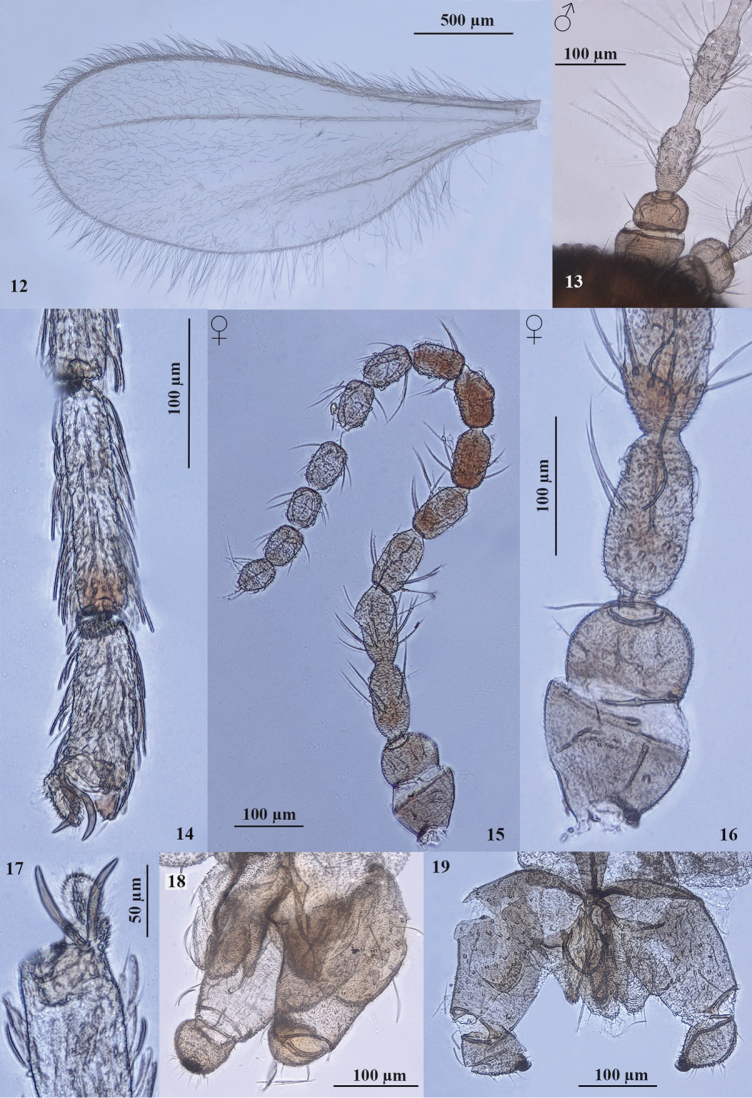
*Rhopalomyia protrahenda* comb. n. **12** Female, wing **13** male, first flagellomeres **14** female, fifth tarsomeres **15** female, antenna **16** female, first flagellomeres **17** female, claws and empodium **18** male terminalia, lateral view **19** frontal view.

**Female** (Figs 1–4, 7–9, 12, 14–17). Body size: 1.8–2.2 mm; wing length 1.6–1.8 mm; wing width 0.68–0.70 mm. Freshly emerged females have head and thorax brown and abdomen orange–reddish. Head with large holoptic eyes, eye bridge about 5 ommatidia long at vertex. Ommatidia relatively small and do not touch. Mouthparts extremely reduced. Frontoclypeus with 18–20 setae. Palpus consists of one short, elliptical segment covered with microtrichia. Antennae: scape obconical, pedicel globular, usually 12 flagellomeres, 1st and 2nd connate, ovoid, without necks, densely covered with microtrichia, each with several long setae; of seven females, five had 12 and two 13 flagellomeres, in accordance with information in De Stefani (1919), who reported that about one third of the females had 13 flagellomeres. Flagellomeres gradually decrease in size to end of antenna; circumfila poorly visible, comprising two transverse and one longitudinal band.

Thorax. Wing with R_5_ joining costa at wing apex, well visible only in basal two thirds; Cu barely visible; anterior wing margin with shorter setae, posterior wing margin with long setae; wing surface covered with short setae. Legs long, covered with short hairs. All legs with simple claws, empodia as long as claws.

Abdomen: Second to sixth segments broad, seventh segment narrow; 8th tergite entire. Ovipositor protrusible, cerci fused, ovoid, setose and setulose; hypoproct small, rounded, setulose, visible only in dorsoventral view.

**Male** (Figs 5, 6, 11, 13, 18–19). Body size: 1.7–1.9 mm; wing length 1.7–1.9 mm; wing width 0.75–0.80 mm.

Antennae: scape obconical, pedicel globular, usually 13 flagellomeres, 1st and 2nd connate; each flagellomere composed of basal node and distal neck; neck about one half of node. Of three males, one male had 12 and two males had 13 flagellomeres.

Terminalia: gonocoxites thick, completely setulose, with several long setae; gonostyli ovoid, short and thick, completely setulose, covered with setae, at the tip with large, brown, beak–formed curved tooth; cerci broad, lobes rounded, deeply separated, setulose; hypoproct slender, incised; mediobasal lobes setulose, apically with several long setae; aedeagus rounded at tip, as long as cerci.

Other morphological characters as in female.

**Larva.** Body size: 1.2–1.8 mm long, 0.39–0.55 mm broad, cream colored; integument covered densely with very small spiculae; without spatula sternalis, without apparent papillae on ninth abdominal segment.

**Pupa** (Figs 10, 27, 28). Body size: 1.5–2.2 mm long, 0.6–0.9 mm broad; head and thorax brown, abdomen orange-red, integument with very small spiculae. Antennal horns small, dark brown; cephalic papillae bulbous with short setae, face with one small horn; prothoracic spiracle small, in the form of a small bulb. Pupal exuviae hyaline.

**Figures 20–29.**
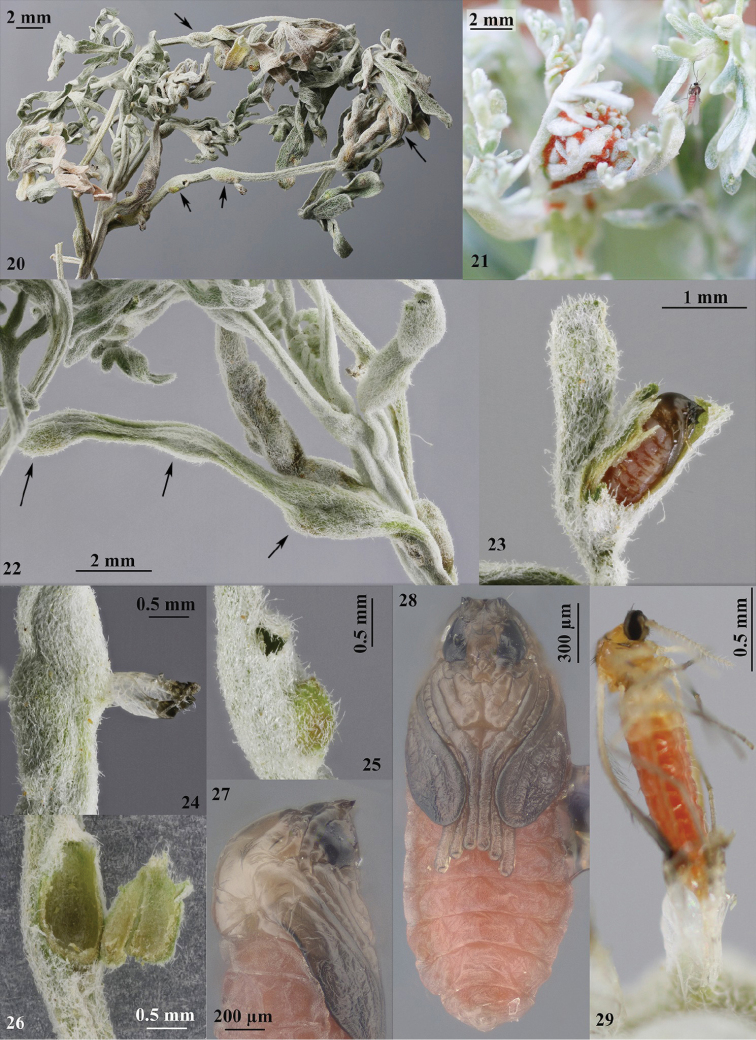
**20** *Artemisia arborescens* covered with galls **21** female of *Rhopalomyia protrahenda* next to apical leaves of the host plant covered with reddish mass of eggs **22** galls, detail **23** dissected gall showing a pupa **24** exuviae protruding from a gall **25** adult emergence hole **26** detail of a leaf gall showing the chamber and thin wall **27** pupa, lateral view **28** pupa frontal view **29** female emerging from gall.

#### Biology

(Figs 20–26, 29). Larvae cause small galls on leaves, leaf stalks and stems of *Artemisia arborescens*. The galls are small hypertrophies of various forms. Only one larva develops in each gall. If galls are abundant, they may cause considerable damage (stunting) of the whole host plant. Larvae pupate in the galls. Several overlapping generations develop per year. New galls may be found on new buds from January onwards, with the emergence of adults until late April and early May. De Stefani (1919) stated that pupae of the last generation use their antennal horns to break out of the gall and drop to the soil, where they remain until the next winter, when the adults emerge. However, his observation is improbable and it is possible that this species diapauses in the larval stage, similar to other *Rhopalomyia* spp. De Stefani (1919) also stated that females of this species are characterized by high fertility and are able to lay a very high number of eggs on leaves of the host plant; he recorded a mean of 127 eggs per female (78–177) (see also Fig. 21).

#### Material examined and neotype designation.

The neotype of *Rhopalomyia protrahenda* is designated here: female, Italy, Sicily, Raffo Rosso, reared from small leaf gall on *Artemisia arborescens*, 18.III.2008, leg. G. Cerasa, microscope slide number 9571, in the collection of National Museum, Prague, Czech Republic (coll. Marcela Skuhravá).

#### Other material.

5♀, 3♂, Italy, Sicily, Palermo, locality Raffo Rosso, galls on *Artemisia arborescens* 3.III.2008, emerged 10–25.III.2008; 3♀, 2♂ same data, emerged 13.II.2012, leg. G. Cerasa; 4♀, 3♂, Italy, Sicily, Cinisi, locality Costa del Furi (Palermo), galls on *Artemisia arborescens* 11.III.2015, emerged 14–16.III.2015, leg. G. Cerasa; 5♀, 3♂, Italy, Sicily, Palermo, locality Raffo Rosso, galls on *Artemisia arborescens* 15.II.2016, emerged 16–25.II.2016, leg. G. Cerasa (in ethanol, BMUP); 2♂, 2♀ (neotype included) mounted (on microscope slides in Canada Balsam, coll. Skuhravà, NMPC); 4♂, 13♀, 6 larvae, 10 pupal exuviae (in Hoyer´s medium, NMPC).

#### Distribution.

The host plant *Artemisia arborescens* is native to the Mediterranean. It is an erect evergreen perennial, with masses of finely–divided aromatic, silvery–white leaves and single–sided sprays of yellow flowers. The plant occurs from the Iberian Peninsula to Israel, the Italian Peninsula, Sicily, the Balkan Peninsula, Turkey and North Africa, and is naturalized in France. *Rhopalomyia protrahenda* has been found only in Sicily.

#### Taxonomic position.

Detailed study of morphological characters of larvae, pupae and adults obtained from galls on *Artemisia arborescens*, found not far from the type locality in Palermo where De Stefani collected galls in the past, revealed that the causer of these galls, described as *Pumilomyia protrahenda* by De Stefani (1919), belongs in the genus *Rhopalomyia* Rübsaamen 1892, and according to the key to the genera of Cecidomyiidae (Skuhravá 1997) it belongs to the tribe Rhopalomyiini and not the Asphondyliini. The genus *Pumilomyia* is therefore synonymized here under *Rhopalomyia* because none of its morphological characters differentiates it from *Rhopalomyia*. De Stefani (1919) incorrectly assessed morphological characters of adult gall midges, mainly the number of antennal flagellomeres (12), shape of female flagellomeres and of ovipositor – and placed his new genus *Pumilomyia* in the Asphondylariae.

The female of *Rhopalomyia protrahenda* has mostly 12 antennal flagellomeres as is typical for all species of *Asphondylia* but the female flagellomeres of *Rhopalomyia protrahenda* are oval and not cylindrical as in females of *Asphondylia*. The female of *Rhopalomyia protrahenda* has the terminal part of ovipositor slender ending with soft, fused cerci, not aciculate and strongly sclerotized as in females of *Asphondylia*.

The genus *Rhopalomyia* Rübsaamen, 1892 is characterized by the combination of the following characters: reduced mouthparts with 1 or 2 segmented palpi, rarely 3; 12-25 flagellomeres; male flagellomeres with long necks, female flagellomeres with short necks; wing with R_5_ reaching Costa nearly at wing apex; simple or toothed tarsal claws; male genitalia with stout gonocoxites and short, completely setulose gonostyli, with large claw; mediobasal lobes sheath aedeagus, cerci broad, hypoproct slender; female 8th tergite entire; ovipositor long, soft, not distinctly sclerotized; cerci fused. Larvae of most species do not have a spatula sternalis and their papillae are barely visible; pupae usually have strongly developed antennal horns.

The genus *Rhopalomyia* includes 267 species worldwide (Gagné and Jaschhof 2014). Larvae induce galls on stems, buds and leaves of host plants almost exclusively of the family Asteraceae. Galls of the same species often occur on several organs of the same host plant species. Alternation of plant organs is connected with the development of several generations per year (e. g., *Rhopalomyia tanaceticola* (Karsch 1879) on *Tanacetum vulgare* and *Rhopalomyia millefolii* (Loew 1850) on *Achillea millefolium*). Pupation takes place inside the galls. Species included in the genus are relatively uniform morphologically and show great reduction in taxonomically useful characters. Dorchin et al. (2009) considered that the best means to distinguish among species remains the morphology and structure of their galls together with the identity of the host plant.

*Rhopalomyia* is relatively species–rich in central, southeast and western Europe, where it contains 38 species. The number of species decreases markedly towards the south. No species were found in the islands of the Mediterranean Sea (other than Sicily) in the past. It is interesting that *Rhopalomyia protrahenda* is the only species of the genus *Rhopalomyia* occurring on a Mediterranean island (Sicily). Many *Rhopalomyia* species have been found to occur in Central Asia, mainly in Kazakhstan (Fedotova 2000, Skuhravá and Skuhravý 2010). The tribe Rhopalomyiini, as defined recently by Gagné in Gagné and Jaschhof (2014), includes two genera: the large genus *Rhopalomyia* with 267 species distributed in all zoogeographic regions and associated mainly with various host plants of the family Asteraceae, and the smaller genus *Psectrosema* with 26 species associated with host plants of the genus *Tamarix* (Tamaricaceae), occurring only in the Palaearctic Region. Only two species belonging to this tribe occur in Sicily: *Rhopalomyia protrahenda* (De Stefani 1919) and *Psectrosema tamaricis* (De Stefani 1902), both found in the Botanical Garden of Palermo by the Sicilian entomologist Teodosio De Stefani.

## Supplementary Material

XML Treatment for Rhopalomyia protrahenda
